# Polymer Selection for Esomeprazole Solid Dispersions: Drug–Polymer Compatibility Assessed by Forced Degradation, ATR-FTIR, and DFT Calculations

**DOI:** 10.3390/polym18212655

**Published:** 2026-10-26

**Authors:** Edgar Ahumada-Herazo, Juan R. Urrego A., Claudia Baena-Aristizábal, Juan José Carrascal, Carlos-A. Bernal Rodríguez

**Affiliations:** 1 Research Group of Pharmaceutical, Cosmetic and Food Technology (GITFCA), Facultad de Ciencias Farmacéuticas, Universidad de Cartagena, Cartagena 130014, Colombia; jurregoa1@unicartagena.edu.co (J.R.U.A.); cbaenaa@unicartagena.edu.co (C.B.-A.); cbernalr@unicartagena.edu.co (C.-A.B.R.); 2 Departamento de Ciencias de la Vida, Facultad de Ciencias de la Vida, Universidad Nacional de Colombia—Sede Medellín, Medellín 050034, Colombia; jjcarrascals@unal.edu.co

**Keywords:** esomeprazole, HPMCAS, HPMCP, Eudragit L100-55, FTIR, forced degradation, density functional theory, drug–polymer compatibility, solid dispersion, non-covalent interactions

## Abstract

Esomeprazole (ESO) is susceptible to degradation under stress conditions, making polymer selection important for stable solid dispersions. This study evaluated ESO compatibility with five polymers: three grades of hydroxypropyl methylcellulose acetate succinate (HPMCAS-LMP, HPMCAS-MMP, and HPMCAS-HMP), hydroxypropyl methylcellulose phthalate (HPMCP-55), and methacrylic acid–ethyl acrylate copolymer (1:1; Eudragit^®^ L100-55). Esomeprazole, polymers, and binary mixtures were stored at room temperature and subjected to forced thermal degradation at 60 °C for 16 days. Spectral changes were assessed by visual inspection and attenuated total reflectance–Fourier transform infrared (ATR-FTIR) spectroscopy using the fractional change index (FCI), normalized root-mean-square deviation (NRMSD), and relative maximum spectral difference (relative Max Δ). Density functional theory (DFT) calculations examined interaction energies, non-covalent interactions (NCIs), frontier molecular orbitals, and HOMO–LUMO-based reactivity descriptors. Combining quantitative ATR-FTIR analysis under thermal stress with DFT provides complementary experimental and molecular-level insights into ESO–polymer interactions, including an experimental comparison of three HPMCAS grades. The ESO–HPMCAS-MMP mixture showed the smallest spectral deviations. DFT indicated favorable interactions and relatively low chemical reactivity for the modeled ESO–HPMCAS complex. These findings support the preliminary consideration of HPMCAS-MMP for further formulation studies, although additional evidence is needed to establish comparative chemical compatibility and stability.

## 1. Introduction

Proton pump inhibitors (PPIs) are a class of acid-suppressing drugs characterized by a substituted benzimidazole–pyridine chemical structure [1]. They are widely used in the treatment of acid-related gastrointestinal disorders, including peptic ulcers, Zollinger–Ellison syndrome, gastroesophageal reflux disease (GERD), and Barrett’s esophagus [2]. Among PPIs, esomeprazole is widely used for the treatment of acid-related gastrointestinal disorders. However, its pharmaceutical development presents several challenges. Like other PPIs, esomeprazole is sensitive to acidic environments, light, temperature, oxidative conditions, and the presence of other salts [3,4,5,6]. These factors can affect its chemical stability, creating challenges during the manufacture, transportation, and storage of pharmaceutical dosage forms. In addition, the low aqueous solubility of esomeprazole can limit its dissolution and, consequently, its bioavailability [7,8,9]. This limited aqueous solubility is partly associated with the solid-state properties of PPIs and the stability of their crystalline forms [10,11]. Several pharmaceutical formulation strategies have therefore been proposed to address these challenges, including enteric-coated minitablets with optimized disintegrant content, cyclodextrin inclusion complexes, nanosuspensions for intravenous administration, and amorphous solid dispersions (ASDs) [12,13,14,15,16]. Among these approaches, ASDs are particularly promising because they can modify the crystalline state of the drug while enhancing its dissolution and potentially improving its bioavailability [17]. However, despite their advantages, ASDs present several formulation and stability challenges, including physical instability, drug–polymer phase separation, moisture sensitivity, limited drug loading, and difficulties in maintaining supersaturation during dissolution. Among these, recrystallization of the amorphous drug during storage remains a major concern, as it can compromise the enhanced solubility and dissolution performance provided by the ASD, making the selection of a suitable polymer essential for maintaining the amorphous state and protecting the active pharmaceutical ingredient (API) [18].

Previous studies have explored solid dispersion systems of esomeprazole and its salts using different polymeric carriers and preparation techniques. For example, Esomeprazole zinc has been formulated with polyethylene glycol (PEG 4000 and PEG 6000) and polyvinylpyrrolidone (PVP K30) using solvent-based methods [19,20], resulting in an amorphous drug state and improved dissolution. In addition, solid dispersions of esomeprazole magnesium have also been prepared using hydroxypropyl methylcellulose (HPMC), with the incorporation of alkalizers to improve the stability of the acid-labile drug in gastric conditions [21].

Despite these previous studies, the compatibility of esomeprazole with polymers containing different functional groups remains insufficiently characterized, particularly for polymers considered for solid dispersion development. Previous studies have shown that polymer composition and acidic functional groups can influence drug–polymer interactions and the stability of proton pump inhibitors [22,23,24]. However, the comparative spectral responses of different HPMCAS grades in combination with esomeprazole under the same defined thermal-stress conditions have not been systematically established. In particular, comparing HPMCAS-LMP, HPMCAS-MMP, and HPMCAS-HMP can help determine whether the observed spectral changes differ among grades under the conditions investigated.

Accordingly, HPMCAS, HPMCP, and Eudragit^®^ L100-55 were selected as representative pharmaceutical polymers with different chemical functionalities and established applications in solid dispersion systems [25,26,27,28]. In these polymers, acidic functional groups remain predominantly protonated under acidic conditions and become increasingly ionized as the pH rises in the intestinal environment, facilitating polymer dissolution in the small intestine [22,29,30]. However, the presence of acidic functional groups may also influence the stability of acid-labile PPIs [23,24].

Beyond their enteric functionality, polymer selection for hot melt extrusion (HME) also requires consideration of thermal and rheological properties, including glass transition temperature (Tg), thermal stability and degradation onset, softening or melt behavior, melt viscosity, and the resulting processing window between polymer softening and thermal degradation [27]. These characteristics are particularly relevant for thermolabile and hydrolysable drugs such as esomeprazole, for which excessive processing temperatures, prolonged residence times, or unfavorable shear conditions may increase the risk of drug degradation. Thus, the distinct chemical structures and physicochemical properties of HPMCAS, HPMCP, and Eudragit^®^ L100-55 provide a relevant basis for investigating their drug–polymer interactions and their potential applicability to HME-based solid dispersion development.

Therefore, the present study aimed to preliminarily evaluate the compatibility of esomeprazole with three pharmaceutical polymers—HPMCAS, HPMCP, and Eudragit^®^ L100-55—as a first step toward their subsequent evaluation for the development of an esomeprazole solid dispersion by HME. The study focused on assessing spectral changes following thermal stress using quantitative ATR-FTIR descriptors and exploring potential drug–polymer molecular interactions through complementary computational approaches. Quantitative ATR-FTIR analysis enables the comparison of changes in measured spectral profiles, whereas molecular docking and density functional theory (DFT) provide complementary perspectives on these interactions, allowing potential molecular associations to be explored alongside the energetic and electronic factors that may contribute to their stability [31]. By combining these approaches, the study aimed to establish a rationale for polymer selection and guide subsequent formulation studies, including the evaluation of thermal behavior, rheological properties, and processing performance during hot-melt extrusion (HME).

## 2. Materials and Methods

### 2.1. Materials

Esomeprazole, used as the active pharmaceutical ingredient (API), was supplied by Nifty Labs (Hyderabad, Telangana, India). The pharmaceutical polymers employed in the physicochemical compatibility studies included three grades of hydroxypropyl methylcellulose acetate succinate (HPMCAS), namely HPMCAS-LMP (Low Medium Substitution), HPMCAS-MMP (Medium Medium Substitution), and HPMCAS-HMP (High Medium Substitution), together with hydroxypropyl methylcellulose phthalate (HPMCP-55). These polymers were obtained from Shin-Etsu Chemical Co., Ltd. (Tokyo, Japan). Eudragit^®^ L100-55 (methacrylic acid–ethyl acrylate copolymer, 1:1, Mw = 320,000) was obtained from Evonik Industries AG (Darmstadt, Germany).

### 2.2. Experimental Evaluation of Esomeprazole–Polymer Compatibility

Because a spectral change in a binary mixture is meaningful only against the behavior of its isolated components, esomeprazole, the polymers, and their corresponding physical mixtures were processed in parallel and evaluated before and after thermal stress. Forced degradation at 60 °C for 16 days served as the stress condition, and ATR-FTIR provided the analytical readout, subsequently expressed through quantitative spectral change descriptors rather than qualitative comparison. These conditions were selected for preliminary evaluation under thermal stress, considering previous pharmaceutical compatibility studies using elevated temperatures and similar exposure periods [32,33]. The individual procedures are detailed in the following subsections.

#### 2.2.1. Sample Preparation

Individual samples of esomeprazole and each polymer were prepared by weighing 1.0 g of each component. Binary mixtures were prepared at a 1:1 (*w*/*w*) drug-to-polymer ratio by manually homogenizing 500.0 mg of esomeprazole with 500.0 mg of the respective polymer. Each system was divided into two experimental groups: a reference group stored at room temperature and a second group subjected to forced thermal degradation at 60 °C for 16 days. After the storage period, all samples underwent preliminary visual inspection and were characterized by ATR-FTIR spectroscopy.

#### 2.2.2. Fourier Transform Infrared Spectroscopy

Infrared spectra were acquired using a Cary 630 FTIR spectrometer (Agilent Technologies, Inc., Santa Clara, CA, USA) equipped with an attenuated total reflectance (ATR) module. The powder samples were analyzed directly by placing them onto the ATR crystal for spectral acquisition. Spectra were recorded over the range of 4000–650 cm^−1^ at a spectral resolution of 4 cm^−1^, with 64 scans collected per sample. All measurements were performed in duplicate under identical instrumental conditions. The duplicate measurements corresponded to two technical measurements of the same sample preparation and were not independent sample preparations.

#### 2.2.3. Spectral Data Processing

Spectral data processing and statistical analyses were performed using Python (version 3.12.7). The FTIR spectra were exported as transmittance (%T) data. For the reanalysis, transmittance values were converted to absorbance using A = 2 − log10(%T). The spectra were linearly interpolated onto a uniform wavenumber grid with a 2 cm^−1^ interval [34] and baseline-corrected using the asymmetric least-squares method (λ = 10^5^, *p* = 10^−3^, and 12 iterations) [35]. Each spectrum was normalized to unit L2 norm before quantitative comparison [36]. The quantitative analysis was restricted to the 1800–650 cm^−1^ region [37]. No additional smoothing or ATR correction was applied.

Three dimensionless descriptors were calculated to characterize the differences between reference and thermally stressed spectra: the fractional change index (FCI), normalized root-mean-square deviation (NRMSD), and relative maximum spectral difference (relative Max Δ). These descriptors were interpreted as measures of relative spectral change, not as direct measurements of chemical degradation or compatibility.

The FCI was calculated as the integrated absolute difference between the stressed and reference spectra divided by the integrated absolute intensity of the reference spectrum:

(1) $$\mathrm{FCI}\;=\frac{\sum_{i=1}^{n}|A_{\mathrm{stress},i}-A_{\mathrm{ref},i}|\Delta \nu i}{\sum_{i=1}^{n}|A_{\mathrm{ref},i}|\Delta \nu i}$$

where A_stress,i_ and A_ref,i_ are the processed, normalized absorbance values of the stressed and reference spectra at point i, respectively, and Δν_i_ is the local wavenumber interval.

The NRMSD was calculated by normalizing the root-mean-square difference to the root-mean-square intensity of the reference spectrum:

(2) $$\mathrm{NRMSD}=\frac{\sqrt{\frac{1}{n}\sum_{i=1}^{n}{\left(A_{\mathrm{stress},i}-A_{\mathrm{ref},i}\right)}^{2}}}{\sqrt{\frac{1}{n}\sum_{i=1}^{n}A_{\mathrm{ref},i}^{2}}}$$

The relative Max Δ was calculated as the maximum absolute pointwise difference divided by the maximum absolute intensity of the reference spectrum:

(3) RelativeMax Δ=max i|Astress,i−Aref, i|max i|Aref,i|

For each material or binary mixture, the descriptors were calculated for all four possible pairings between the two reference-condition measurements and the two thermally stressed measurements. The mean and standard deviation across these four pairings were used to describe the observed variation. The FCI was also calculated between measurements obtained under the same condition to provide a descriptive estimate of within-condition spectral variability. These analyses were descriptive because the duplicate measurements corresponded to technical measurements of the same sample preparation rather than independent sample preparations. Therefore, the observed standard deviations do not represent between-preparation variability, and no inferential conclusions were drawn.

### 2.3. Quantum Mechanical Modeling

Esomeprazole and three pharmaceutical polymers—hydroxypropyl methylcellulose acetate succinate (HPMCAS), hydroxypropyl methylcellulose phthalate (HPMCP), and Eudragit^®^ L100-55—were constructed using GaussView 6.0.16 (Gaussian, Inc., Wallingford, CT, USA) [38]. The HPMCAS model comprised a finite oligomer of three anhydroglucose units, corresponding to a degree of polymerization of three, with a nonuniform substitution. The fragment contained methoxy, acetyl, 2-methoxypropyl, 2-acetoxypropyl, and succinoyl substituents, with both succinoyl groups retaining protonated terminal carboxylic acid groups. The HPMCP model comprised two anhydroglucose units, corresponding to a degree of polymerization of two, and contained 2-hydroxypropyl substituents and a phthalate group attached through an ester linkage, with a terminal carboxylic acid group. The Eudragit^®^ L100-55 model consisted of a fragment containing four methacrylic acid units and four ethyl acrylate units along a carbon–carbon backbone, incorporating free carboxylic acid and ethyl ester groups. The three polymer fragments were designed to contain approximately 110–130 atoms to maintain comparable molecular sizes and approximate interaction surfaces for the computational analysis. All polymer fragments were modeled with a total charge of zero and a singlet electronic state with a multiplicity of one.

The molecular structures of the polymer fragments and esomeprazole were independently optimized using density functional theory (DFT) at the B3LYP/6-31G(d) level [39] in Gaussian 16, Revision C.01 (Gaussian, Inc., Wallingford, CT, USA) [40], until SCF convergence was achieved. Geometry optimizations were followed by frequency calculations to verify that the optimized structures corresponded to a true minimum on the potential energy surface. No imaginary frequencies were observed for the individual polymers and the esomeprazole.

The optimized structures of the polymer fragments and esomeprazole were used as input for molecular docking calculations performed with AutoDock 4.2 (The Scripps Research Institute, La Jolla, CA, USA) [41]. Molecular docking with esomeprazole was performed using AutoDock4 with the Lamarckian genetic algorithm (LGA). For each polymer–ligand system, 100 independent docking runs were conducted using a population size of 150 individuals, a maximum of 2,500,000 energy evaluations per run, and a maximum of 27,000 generations. Gasteiger partial charges were assigned to both esomeprazole and the polymer models. The polymers were treated as rigid receptors during docking. The grid spacing was set to 0.0375 nm, and the grid dimensions were adjusted to encompass the complete structure of each polymer. The docking grid was configured with dimensions of 60 × 60 × 60 grid intervals, corresponding to a spatial extent of approximately 2.25 × 2.25 × 2.25 nm. The resulting docking poses were ranked according to their predicted binding energies, and poses with the lowest (most negative) binding energies were selected for subsequent analysis of esomeprazole–polymer interactions [42,43].

The selected docked complexes were subsequently reoptimized using the long-range corrected hybrid functional ωB97X-D in conjunction with the 6-31++G(d,p) basis set [44,45] in Gaussian 16, until SCF convergence was achieved. Dispersion interactions were explicitly included through the ωB97X-D functional. Frequency calculations were performed to confirm that the optimized structures corresponded to local minima on the potential energy surface, with no imaginary frequencies observed.

The counterpoise method was applied to the two-fragment system comprising the polymer and esomeprazole to correct for basis set superposition error (BSSE), allowing the calculation of BSSE-corrected interaction energies [46]. Gibbs free energies of complex formation (ΔG) were obtained from the thermochemical analysis using the BSSE-corrected electronic energies.

To characterize the intermolecular interactions responsible for complex stabilization, non-covalent interaction (NCI) analysis was carried out using Multiwfn 2026.10.18 (Beijing Kein Research Center for Natural Sciences, Beijing, China) [47,48]. The reduced density gradient (RDG) and the sign(λ_2_)ρ descriptor were used to identify attractive interactions (hydrogen bonds, van der Waals interactions) and steric repulsion.

Finally, electronic reactivity descriptors were calculated from the frontier molecular orbital energies of the optimized structures. The descriptors included the HOMO and LUMO energies, energy gap (ΔE), ionization potential (I), electron affinity (A), chemical hardness (η), chemical potential (μ), electronegativity (χ), and electrophilicity index (ω), according to Koopmans’ approximation [49,50,51].

## 3. Results and Discussion

### 3.1. Compatibility Studies

Visual inspection of the samples before and after thermal treatment at 60 °C for 16 days allowed the identification of macroscopic changes potentially associated with physical or chemical modifications induced by the forced degradation conditions. Although this type of assessment does not provide direct evidence of incompatibility between the active pharmaceutical ingredient and the excipients, it represents a preliminary approach for evaluating the behavior of the materials under thermal stress.

Esomeprazole exhibited a change in coloration after thermal treatment (Table 1), changing from a white powder to a slightly beige coloration. This observation is consistent with the reported susceptibility of esomeprazole to heat, moisture, and oxidative conditions [52]. Therefore, the observed color change may indicate the occurrence of initial degradation or chemical transformation of the active pharmaceutical ingredient. A similar behavior was reported by Patel et al. [53] in a drug–excipient compatibility study of esomeprazole, in which the API exhibited a slight color change from off-white to light yellow under 40 °C/75% RH conditions, both under open and closed storage.

The polymers HPMCAS-LMP, HPMCAS-HMP, HPMCAS-MMP, HPMCP-55, and Eudragit^®^ L100-55 showed no appreciable changes in color, texture, or surface appearance following thermal stress (Table 1). The corresponding binary mixtures of esomeprazole with each of these polymers exhibited a similar overall behavior: in all cases, a slight beige or yellowish coloration was observed after thermal stress, comparable to that observed for the pure API, while the texture and powder distribution remained similar to those of the reference samples.

These observations suggest that the macroscopic changes were primarily associated with the response of esomeprazole to thermal treatment rather than with evident physical changes arising from specific interactions with the polymers. Nevertheless, the absence of macroscopic changes does not exclude molecular-level interactions or chemical incompatibilities, which must be further evaluated using complementary analytical techniques.

### 3.2. Fourier Transform Infrared Spectroscopy

The FTIR spectra of the individual compounds were obtained; esomeprazole exhibited some of the most pronounced spectral variations among the compounds studied (Figure 1A). The main modification was observed in the region approximately between 3600 and 2800 cm^−1^, where the stressed sample exhibited an increase in transmittance, reflected as positive signals in the difference spectrum. This region includes, among others, the stretching vibrations of N–H and C–H bonds; in particular, the band around 2970 cm^−1^ has been associated with the asymmetric C–H stretching of aromatic groups [54]. Changes were also identified in the fingerprint region (1600–650 cm^−1^), which contains several characteristic bands of the esomeprazole structure. These include the signals around 1612 and 1588 cm^−1^, associated with C=N stretching in the benzimidazole system, and the band near 1569 cm^−1^, attributed to C=N stretching in the pyridine ring. Likewise, the signals near 1409 cm^−1^ correspond to N–H bending, whereas the bands around 1228 and 1199 cm^−1^ have been associated with C–O stretching of the methoxy groups. The signal near 1076 cm^−1^ is particularly relevant because it corresponds to S=O stretching of the sulfoxide group, a characteristic structural feature of esomeprazole [11].

The spectra of Eudragit^®^ L100-55 were similar before and after thermal stress. According to previous studies [55], the band corresponding to C=O stretching in Eudragit^®^ L100-55 occurs at approximately 1696 cm^−1^, whereas the C–O–C stretching vibration is observed at approximately 1156 cm^−1^. In the present study, the observed differences were of low magnitude and were mainly concentrated in the region associated with C=O stretching vibrations of the carbonyl groups, around 1696–1730 cm^−1^, as well as in some bands in the fingerprint region corresponding to C–O–C and C–O vibrations (Figure 1B) [55]. The difference spectrum predominantly showed low-amplitude negative signals, indicating slight variations in band intensity without relevant shifts in the main characteristic bands. No new absorption bands were observed, and the main spectral bands retained their positions after thermal stress, with the copolymer maintaining its FTIR profile under the evaluated conditions. However, these observations alone do not establish the absence of chemical degradation.

The HPMCAS-MMP grade exhibited a high degree of preservation of its spectral profile. The spectra obtained before and after thermal treatment practically overlapped across the entire spectral range. According to previous studies, HPMCAS exhibits characteristic bands around 3400 cm^−1^, attributed to O–H stretching; around 1750 cm^−1^, corresponding to C=O stretching; between 1400–1350 cm^−1^, associated with C–O–C vibrations; and around 1064 cm^−1^, related to C–O stretching of cyclic ether groups [56,57]. In the present study, the difference spectrum showed only small fluctuations around the baseline, with slightly more pronounced variations between 2200 and 1900 cm^−1^ and a small contribution around 1000 cm^−1^ (Figure 1C). No significant shifts in the characteristic polymer bands or appearance of new absorptions were observed, and the spectral profile of HPMCAS-MMP remained essentially unchanged after thermal treatment.

The FTIR spectra of HPMCP-55 showed slightly greater differences compared with those observed for the HPMCAS grades (Figure 1D). The difference spectrum exhibited negative signals distributed mainly between 1750 and 900 cm^−1^, coinciding with regions associated with the carbonyl groups and C–O–C vibrations of the polymer [55]. Although intensity variations were evident, no formation of new bands or significant shifts in the main absorption bands were detected. These findings indicate changes in the measured spectral profile; however, the observed intensity variations alone do not establish whether they arise from conformational changes, other physical changes, or chemical degradation.

The HPMCAS-HMP grade exhibited a similar spectral behavior. The spectra obtained before and after thermal treatment showed a high degree of overlap (Figure S1). The differences were mainly limited to small variations in intensity, with no significant shifts or new absorption bands. The difference spectrum showed minor fluctuations around the baseline, particularly in the fingerprint region, while the characteristic bands of the polymer remained essentially unchanged.

The HPMCAS-LMP grade also exhibited a similar spectral response (Figure S2). The changes were mainly limited to the fingerprint region, where the difference spectrum showed moderate increases around 1700 cm^−1^ and between 1200 and 900 cm^−1^, corresponding mainly to C=O and C–O/C–O–C vibrations of the polymer ester groups [55]. The main bands retained their position and shape, and no new absorptions or significant shifts were observed after thermal treatment.

The esomeprazole + HPMCAS-MMP mixture exhibited relatively small spectral changes after thermal treatment (Figure 2A). The difference spectrum remained close to the baseline across most of the spectral range, with small positive signals around 3000 cm^−1^ and slight negative signals between 1700 and 1400 cm^−1^. No new absorption bands or significant shifts in the main characteristic bands were observed, indicating that the measured spectral profile remained largely preserved under the evaluated thermal-stress conditions. However, these observations alone do not establish the absence of chemical degradation.

The esomeprazole + HPMCAS-LMP mixture exhibited more pronounced spectral differences than the esomeprazole + HPMCAS-MMP mixture (Figure S3). The difference spectrum showed signals distributed across the evaluated range, with greater differences between approximately 1500 and 900 cm^−1^. This region includes contributions from C–O, C–O–C, and carbonyl-associated vibrations of the polymer and potentially overlapping contributions from esomeprazole [11,56]. However, the observed intensity variations, in the absence of clearly identifiable new absorption bands or consistent peak shifts, do not establish specific chemical transformations or degradation processes.

The esomeprazole + HPMCAS-HMP mixture exhibited a high degree of spectral overlap between the two experimental conditions (Figure S4). The difference spectrum showed low-intensity negative signals distributed mainly between 3500 and 900 cm^−1^, with no appearance of new absorption bands. The observed variations were limited to small changes in band intensity, without significant shifts in the main characteristic spectral bands.

The esomeprazole + Eudragit^®^ L100-55 mixture exhibited spectral differences after thermal treatment (Figure 2B). The difference spectrum showed predominantly negative signals between approximately 1700 and 700 cm^−1^, particularly in the fingerprint region, where C–O–C, C–O, and other polymer-associated vibrations occur. However, no clearly identifiable new absorption bands or consistent shifts in the main characteristic bands were observed. These findings indicate changes in the measured spectral profile under the applied thermal-stress conditions but do not establish whether they arise from chemical transformations, physical changes, or measurement-related variability.

The esomeprazole + HPMCP-55 mixture exhibited increases in spectral intensity mainly between 1500 and 650 cm^−1^, with smaller variations around 3000 cm^−1^ (Figure 2C). Although the spectral differences were greater than those observed for the esomeprazole + HPMCAS-MMP mixture, the main characteristic bands retained their positions. These observations indicate changes in spectral intensity, but they are insufficient to establish specific conformational changes or chemical degradation.

Overall, ATR-FTIR provided a comparative assessment of spectral changes in esomeprazole, the individual polymers, and their binary mixtures following thermal stress. Changes in band intensity or the absence of new bands and significant peak shifts cannot, by themselves, confirm chemical degradation or demonstrate chemical stability and drug–polymer compatibility. Nevertheless, the comparison of spectral profiles and the quantitative descriptors used in this study helped identify the systems exhibiting greater or smaller spectral deviations under the tested conditions. These findings provide a preliminary basis for comparing the responses of the investigated systems and prioritizing further studies, but they should be interpreted alongside complementary analytical techniques, particularly chromatographic methods, to establish chemical degradation and assess compatibility more conclusively [58].

#### Spectral Data Processing

The original spectral change indicators were recalculated after converting the transmittance data to absorbance, interpolating the spectra onto a uniform wavenumber grid, applying asymmetric least-squares baseline correction, and normalizing each spectrum to unit L2 norm. The revised analysis used the fractional change index (FCI), normalized root-mean-square deviation (NRMSD), and relative maximum spectral difference (relative Max Δ) to describe changes in the measured spectral profiles. These dimensionless descriptors reduce dependence on the original intensity scale; however, they should not be interpreted as a universal measure of chemical compatibility across chemically different materials. The resulting metrics for the individual components and binary mixtures are summarized in Table 2.

Among the five esomeprazole–polymer mixtures, the esomeprazole–HPMCP-55 mixture exhibited the highest mean FCI (0.1511) and NRMSD (0.1451), together with a relative Max Δ of 0.1311. Its FCI signal-to-noise ratio was 2.74, indicating that its mean spectral difference was greater than the estimated within-condition variability under the applied descriptive analysis. These results identify a comparatively larger spectral change for this mixture, but the spectral differences do not, by themselves, establish a specific chemical transformation or degradation mechanism.

The esomeprazole–Eudragit^®^ L100-55 mixture had a mean FCI of 0.1305, an NRMSD of 0.1305, and a relative Max Δ of 0.1295. However, its FCI standard deviation across the four reference–stress pairings was 0.1052, and its FCI signal-to-noise ratio was 0.59. Thus, the observed difference was accompanied by substantial variation relative to the estimated within-condition signal. These findings indicate that the esomeprazole–Eudragit^®^ L100-55 mixture exhibited measurable spectral changes under the applied thermal stress. The combined metrics provide complementary information on the magnitude and variability of these changes and support a preliminary assessment of drug–polymer interactions under the conditions investigated. However, further studies are needed to establish the implications of these spectral changes for the overall compatibility of the mixture.

The esomeprazole–HPMCAS-LMP and esomeprazole–HPMCAS-HMP mixtures showed mean FCI values of 0.0604 and 0.0543, respectively, with NRMSD values of 0.0589 and 0.0503, and relative Max Δ values of 0.0655 and 0.0463. These measurements indicate relative changes in the processed spectrum, but the magnitude of the differences should be interpreted in relation to the observed within-condition variability.

The esomeprazole–HPMCAS-MMP mixture exhibited an FCI of 0.0463, an NRMSD of 0.0399, and a relative Max Δ of 0.0312. Its FCI signal-to-noise ratio was 1.95. This mixture showed comparatively low spectral deviations among the evaluated mixtures. The observed preservation of its processed spectral profile supports the preliminary consideration of HPMCAS-MMP for subsequent formulation studies but does not establish definitive chemical compatibility or long-term stability.

The individual polymers also exhibited different degrees of spectral variation after thermal stress. Their FCI values ranged from 0.0241 for HPMCAS-HMP to 0.0482 for HPMCP-55. The FCI for esomeprazole alone was 0.0542, with a signal-to-noise ratio of 0.86, indicating that the spectral change was not clearly distinguished from the within-condition variation using this descriptive comparison. The visible color change observed for esomeprazole should be discussed as a macroscopic observation; the present FTIR data do not establish the chemical identity or concentration of any species responsible for that color change.

Taken together, the normalized descriptors do not support a general ranking of HPMCAS, HPMCP, and Eudragit^®^ L100-55 in terms of chemical compatibility. The results instead show grade-dependent spectral behavior within the HPMCAS group. The relative preservation of the esomeprazole–HPMCAS-MMP spectral profile makes this grade a candidate for further investigation under the evaluated thermal-stress conditions but does not prove compatibility. Because the duplicate measurements were technical measurements of the same sample preparation, these findings should be interpreted descriptively and require confirmation using complementary analytical methods.

### 3.3. Free Gibbs Energy and NCIs

The DFT analysis of the drug–polymer complexes indicated that the esomeprazole–HPMCAS complex exhibited the most favorable interaction among the three complexes evaluated, with an uncorrected interaction energy (ΔEint) of −45.33 kcal/mol, followed by the esomeprazole–HPMCP (−30.87 kcal/mol) and esomeprazole–Eudragit^®^ L100-55 (−16.32 kcal/mol) complexes, as shown in Table 3. After applying the counterpoise correction for basis set superposition error (BSSE), the corresponding corrected interaction energies (ΔEint-CP) were −40.82, −27.86, and −14.27 kcal/mol for the esomeprazole–HPMCAS, esomeprazole–HPMCP, and esomeprazole–Eudragit^®^ L100-55 complexes, respectively.

In the esomeprazole–HPMCAS complex, the NCI analysis indicated a specific interaction between the sulfoxide oxygen of esomeprazole and the carboxylic acid group of the succinate moiety. In addition to this hydrogen-bonding interaction, the polymer surface provides a favorable arrangement around the drug, allowing multiple weak attractive contacts to form while limiting steric clashes (Figure 3A). In the esomeprazole–HPMCP complex, esomeprazole formed a hydrogen bond with a hydroxyl group of the polymer, accompanied by some weak attractive contacts. However, the lower interaction energy compared with HPMCAS may be due to a less favorable overall arrangement (Figure 3B). One possible explanation is that establishing the specific hydrogen bond requires a less favorable conformation of esomeprazole or reduces the extent of other favorable contacts, thereby limiting the overall stabilization of the complex.

Finally, the esomeprazole–Eudragit^®^ L100-55 complex exhibited the weakest interaction energy and was characterized predominantly by weak attractive contacts (Figure 3C). Although Eudragit^®^ L100-55 contains functional groups capable of participating in hydrogen bonding, the formation of a specific hydrogen bond did not necessarily increase the overall stability of the complex. If establishing such an interaction requires an unfavorable molecular rearrangement, it could reduce the effective contact surface or introduce additional steric strain, offsetting the energetic benefit of the hydrogen bond.

### 3.4. Electronic Descriptors

For each complex, the HOMO is shown first, followed by the corresponding LUMO. Analysis of the frontier molecular orbitals showed that, in all complexes, the HOMO is predominantly localized on the esomeprazole moiety (Figure 4), with the largest atomic contribution arising from the methoxy oxygen of the pyridine ring. The HOMO energy is related to the ionization potential (I) and, within Koopmans’ approximation, describes the energy required to remove an electron from the system [59]. Therefore, a higher ionization potential corresponds to a greater electron-donating character. However, the localization of the HOMO on the methoxy oxygen should not be interpreted directly as evidence of nucleophilic reactivity or as indicating that this group is a primary site of degradation. In fact, degradation studies of omeprazole using LC–MS have identified degradation products resulting from transformations at other regions of the molecule [60,61]. The localization of the HOMO around the methoxy oxygen may be more relevant to its potential participation in intermolecular interactions, such as hydrogen bonding with the polymer.

The LUMO, on the other hand, is related to the electron affinity (A) and describes the energy associated with electron acceptance [62]. In most of the complexes, the LUMO is predominantly localized on the esomeprazole moiety. The exception was the HPMCP–esomeprazole complex, in which the LUMO is mainly localized on the polymer, particularly around a carbon atom adjacent to the carboxylic group of the phthalate moiety. This localization can be associated with the electronic effects of the neighboring electron-withdrawing groups and the delocalization effects. Nevertheless, the LUMO+1 in this complex was predominantly localized on the esomeprazole moiety. Therefore, although the LUMO is polymer-centered, the next higher-energy unoccupied orbital was predominantly associated with the drug. This orbital distribution raises the possibility that esomeprazole itself is the main contributor to the electronic reactivity of the complexes, rather than covalent bond formation between the drug and the polymers.

The energy gap (ΔE) is a useful descriptor for evaluating the chemical reactivity of interacting molecules in drug-delivery systems. A smaller HOMO–LUMO gap is generally associated with higher chemical reactivity, whereas a larger gap is associated with lower reactivity [63]. The energy gaps of all three esomeprazole–polymer complexes were lower than those of the corresponding isolated polymers and were also lower than that of isolated esomeprazole (Table 4). Since the frontier molecular orbitals were predominantly localized on the esomeprazole moiety, the changes in their energies mainly occur within the drug region. The HOMO of the complexes has a lower energy than the HOMO of isolated esomeprazole, while the LUMO also had a lower energy. Therefore, both frontier orbitals became more stabilized upon complexation, with the decrease in LUMO energy being greater than the decrease in HOMO energy. As a result, the energy difference between the two orbitals decreased, producing the lower ΔE values observed for the complexes. This reduction raises the possibility that the presence of the polymers increases the chemical reactivity of esomeprazole, potentially making the drug more susceptible to chemical transformation.

These findings are also related to chemical descriptors: chemical hardness (η), which represents the resistance of a system to changes in its electron density; chemical potential (μ), which describes the tendency of a system to exchange electron density; electronegativity (χ), which describes the tendency of a system to attract electron density; and the electrophilicity index (ω), which describes the ability of a system to accept electron density [64].

The chemical potential of esomeprazole decreased from −3.608 eV to −3.802, −3.939, and −4.197 eV after complexation with HPMCAS, HPMCP, and Eudragit^®^ L100-55, respectively. Consequently, the electronegativity increased from 3.608 eV for isolated esomeprazole to 3.802, 3.939, and 4.197 eV in the corresponding complexes, while the electrophilicity index increased from 0.037 eV to 0.040, 0.042, and 0.048 eV, respectively. These changes describe a shift in the global electronic properties of esomeprazole toward greater electron-accepting and electrophilic character after complexation.

The chemical hardness (η) of the esomeprazole-HPMCAS, esomeprazole-HPMCP, and esomeprazole-Eudragit^®^ L100-55 complexes were 4.144, 4.008, and 4.005 eV, respectively, compared with 4.189 eV for isolated esomeprazole. Therefore, esomeprazole-HPMCAS complex was the hardest of the three, whereas esomeprazole-Eudragit^®^ L100-55 complex was the softest. The lower hardness of the complexes corresponds to their smaller HOMO–LUMO gaps and means that changes in their electron density require a smaller energy separation between the occupied and unoccupied frontier orbitals. This effect was particularly pronounced for Eudragit^®^ L100-55, although isolated polymer was the hardest of the three, with a hardness of 5.291 eV, its complex with esomeprazole had the lowest hardness of the three complexes (4.005 eV). The hardness of HPMCAS and HPMCP decreased from 4.959 and 4.522 eV in the isolated polymers to 4.144 and 4.008 eV in their complexes, respectively. Thus, complexation substantially modified the electronic response of the polymers and esomeprazole, producing complexes with lower hardness and greater softness than the corresponding isolated polymers. Together with the predominantly drug-centered frontier orbitals, these results raise the possibility that polymer association increases the chemical reactivity of esomeprazole. However, the descriptors alone cannot establish a specific reaction pathway or degradation rate.

When comparing the three supramolecular complexes, the esomeprazole–HPMCAS complex exhibited the largest HOMO–LUMO gap (8.288 eV) and the highest chemical hardness (4.144 eV) among the evaluated complexes. According to the maximum hardness principle (MHP), these descriptors suggest relatively greater electronic stability of the esomeprazole–HPMCAS complex compared with the esomeprazole–HPMCP and esomeprazole–Eudragit^®^ L100-55 complexes. However, given the preliminary nature of the present study, these findings should be considered as an indication of relative electronic stability rather than direct evidence of greater chemical stability.

## 4. Conclusions

The compatibility study showed that the observed color change was mainly associated with the exposure of esomeprazole to the applied stress conditions (60 °C for 16 days). The FTIR analysis provided a comparative assessment of spectral changes in the five polymer systems and their binary mixtures with esomeprazole following thermal stress. Among the mixtures, esomeprazole–HPMCAS-MMP exhibited the smallest spectral deviations according to the fractional change index (FCI), normalized root-mean-square deviation (NRMSD), and relative maximum spectral difference (relative Max Δ). These results support the preliminary consideration of HPMCAS-MMP for further investigation under the evaluated thermal-stress conditions, but do not establish definitive chemical compatibility or long-term stability. The DFT calculations further supported the favorable behavior of the esomeprazole–HPMCAS system, which exhibited the most favorable molecular interactions among the evaluated complexes. Moreover, the HOMO–LUMO energy gap and chemical hardness suggested relatively greater electronic stability and lower chemical reactivity for the esomeprazole–HPMCAS complex compared with the other evaluated complexes. However, the computational findings should be distinguished from the experimental spectral results and do not establish a general experimental stability ranking among HPMCAS, HPMCP, and Eudragit^®^ L100-55. The differences observed among HPMCAS-MMP, HPMCAS-LMP, and HPMCAS-HMP also indicate that the behavior of HPMCAS should be considered grade-dependent rather than generalized to the polymer class. Additional studies using chromatographic analysis of degradation products and long-term stability testing would be necessary to clarify the degradation behavior, assess chemical compatibility more conclusively, and evaluate the suitability of HPMCAS-MMP for the development of esomeprazole solid dispersions. These findings provide a preliminary basis for subsequent formulation studies under conditions relevant to the intended application.

## Figures and Tables

**Figure 1:**
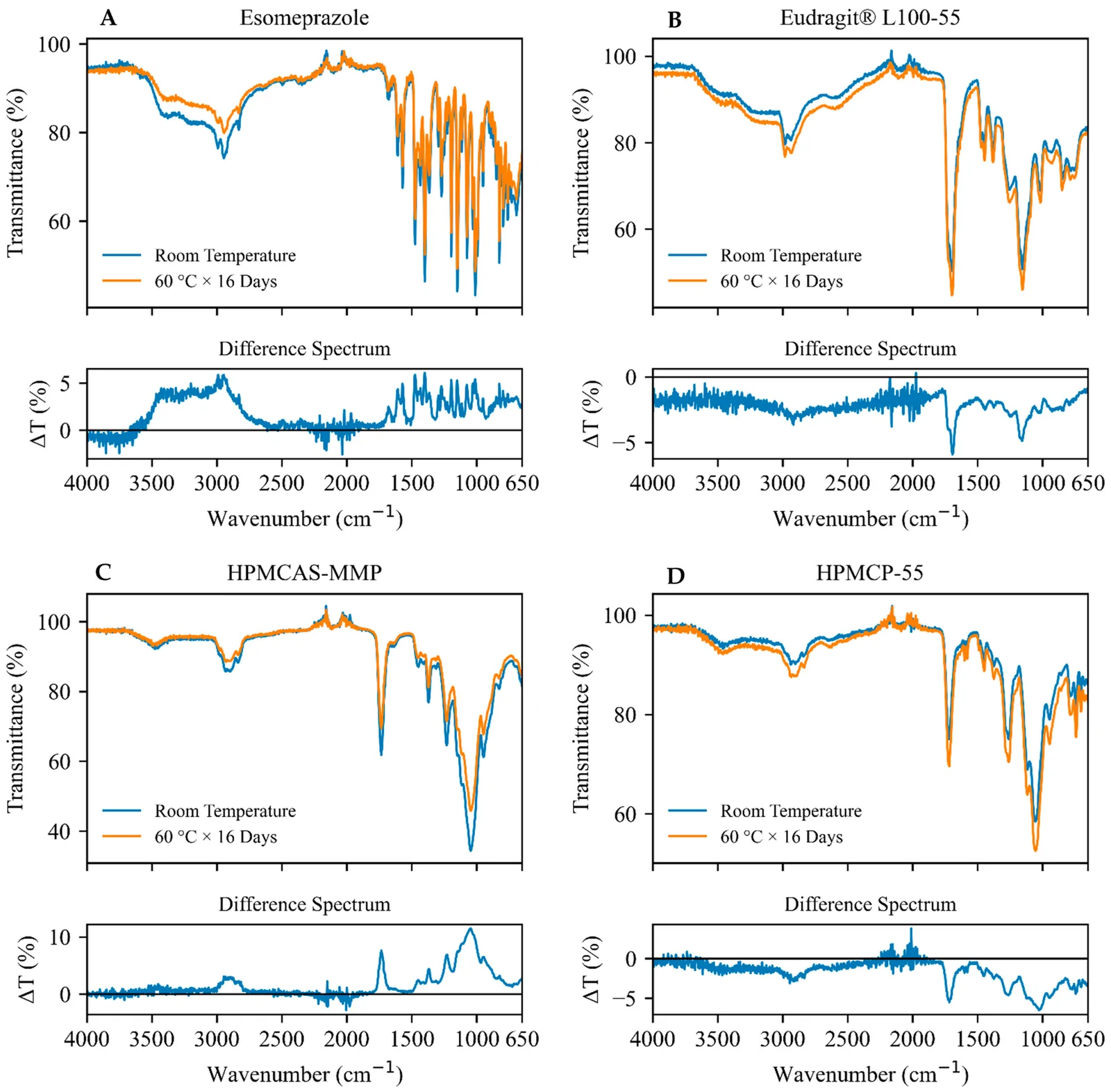
FTIR spectra of the individual components under room-temperature conditions and after thermal treatment at 60 °C for 16 days: (**A**) esomeprazole, (**B**) Eudragit^®^ L100-55, (**C**) HPMCAS-MMP, and (**D**) HPMCP-55.

**Figure 2:**
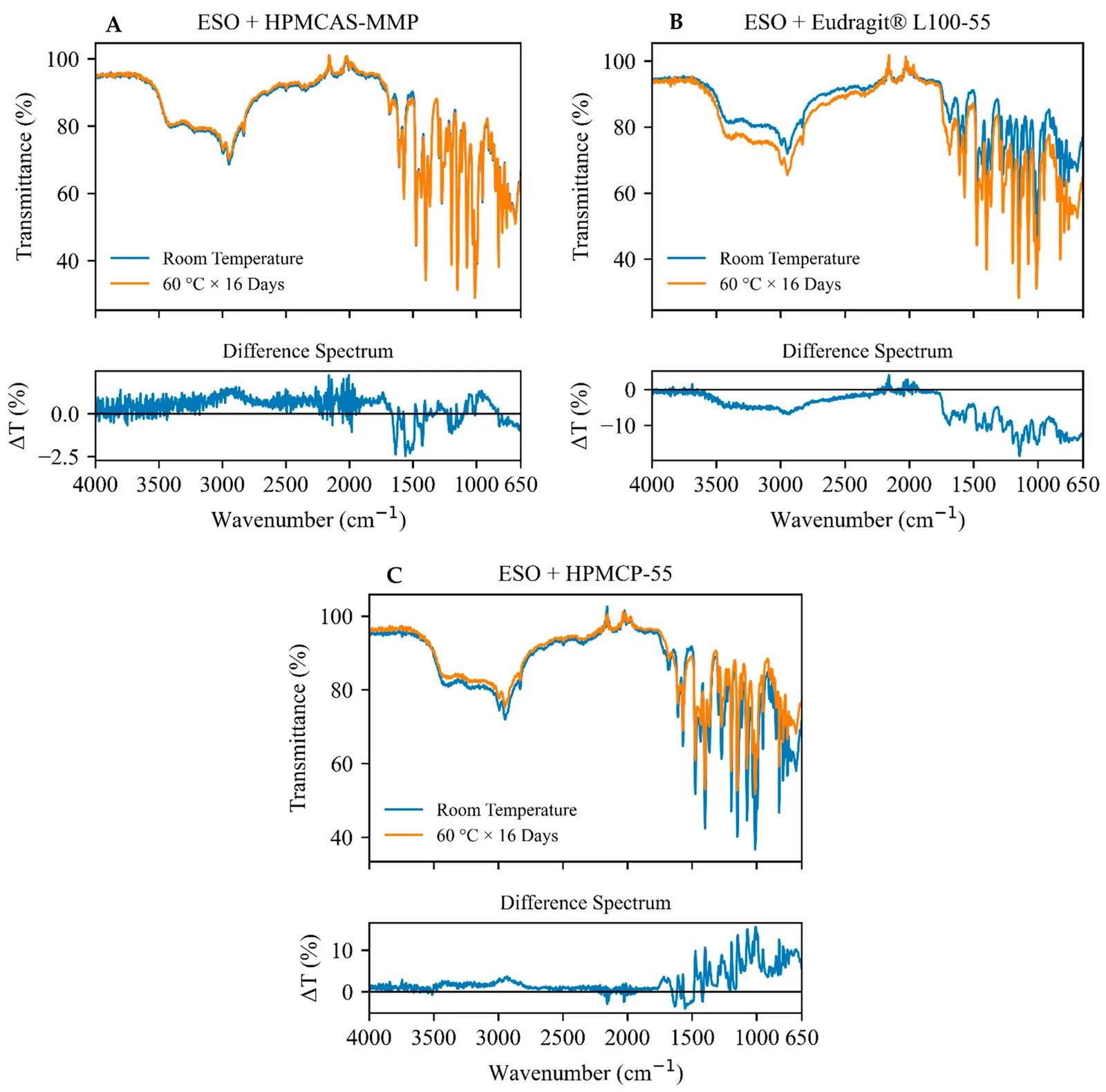
FTIR spectra of the mixtures under room-temperature conditions and after thermal treatment at 60 °C for 16 days: (**A**) esomeprazole–HPMCAS-MMP, (**B**) esomeprazole–Eudragit^®^ L100-55, and (**C**) esomeprazole–HPMCP-55.

**Figure 3:**
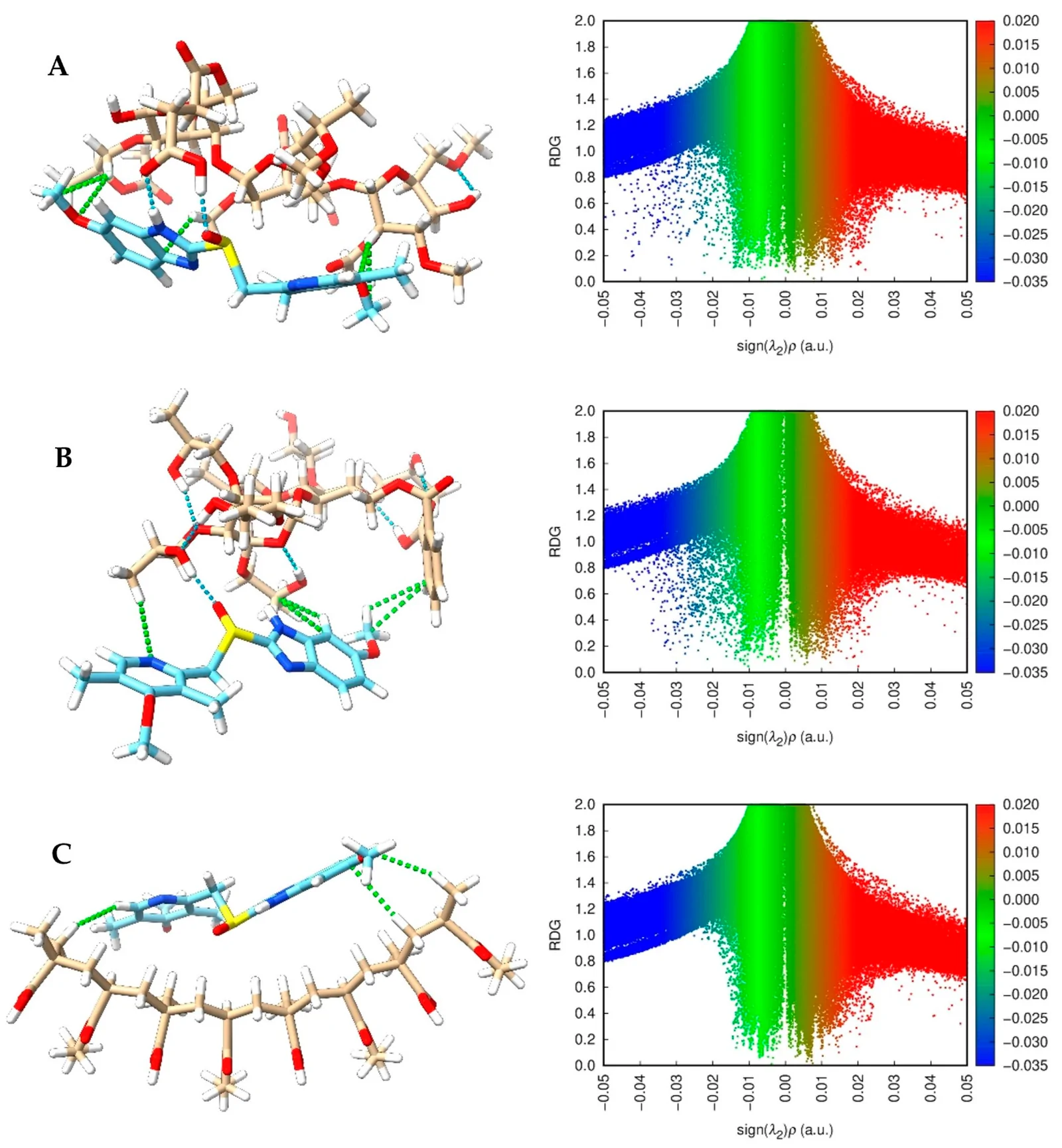
NCIs for (**A**) esomeprazole–HPMCAS, (**B**) esomeprazole–HPMCP, and (**C**) esomeprazole–Eudragit^®^ L100-55. The corresponding reduced density gradient (RDG) plots are presented to the right of each complex. For improved visualization, the NCI isosurfaces were replaced by dotted interaction lines in the molecular structures. Blue dotted lines represent hydrogen-bonding interactions, whereas green dotted lines represent van der Waals contacts. Steric interactions are not displayed.

**Figure 4:**
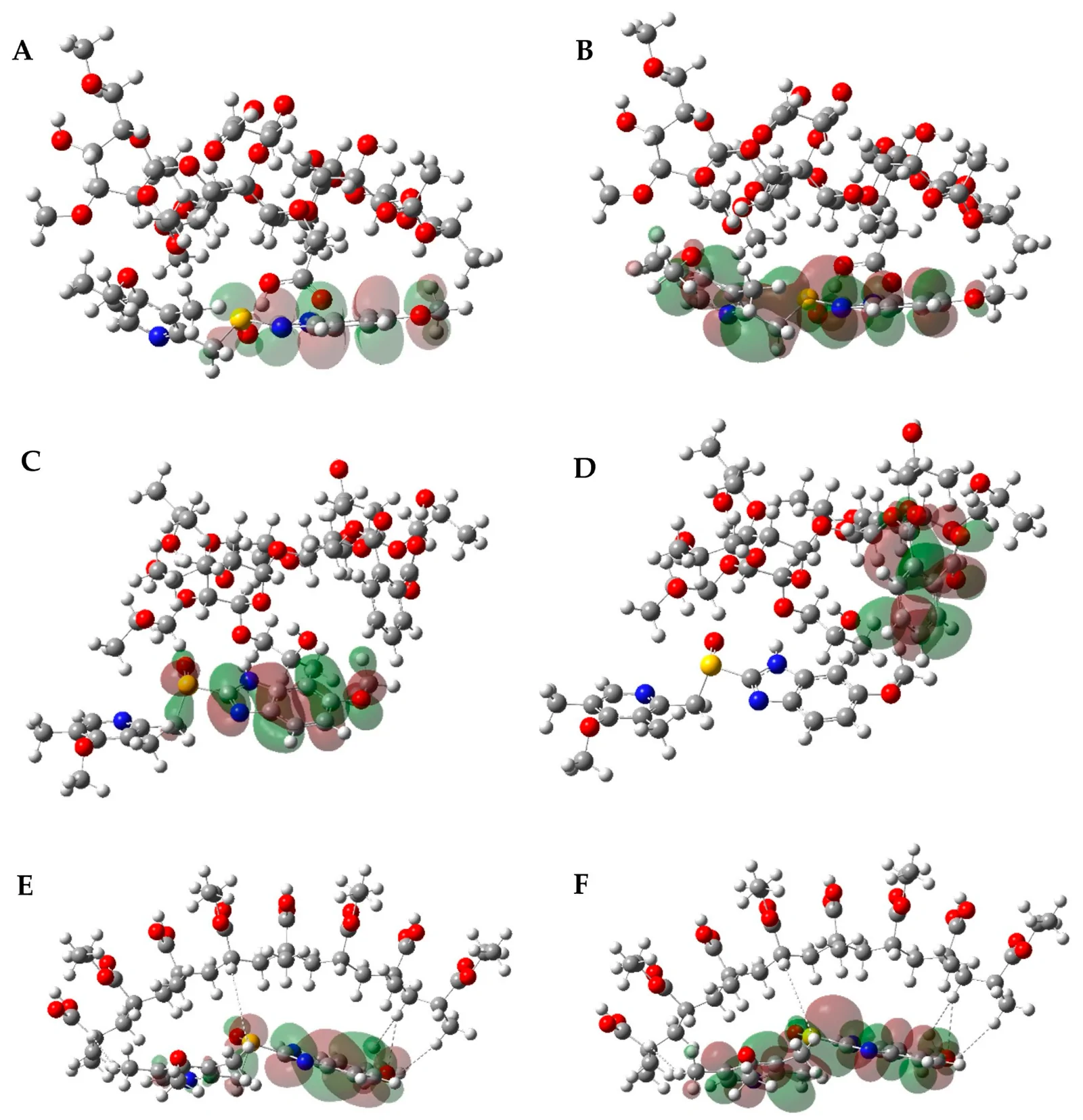
Frontier molecular orbitals of the esomeprazole–polymer complexes. HOMO and LUMO distributions for the (**A**,**B**) esomeprazole–HPMCAS, (**C**,**D**) esomeprazole–HPMCP, and (**E**,**F**) esomeprazole–Eudragit^®^ L100-55 complexes. For each complex, the HOMO is presented first, followed by the corresponding LUMO. The atoms are represented using the conventional color scheme: carbon in gray, hydrogen in white, nitrogen in blue, oxygen in red, and sulfur in yellow.

**Table 1:** Visual appearance of individual components and esomeprazole–polymer mixtures under room-temperature conditions and after thermal treatment at 60 °C for 16 days.

Sample	Room Temperature	60 °C for 16 Days	Evaluation
Esomeprazole	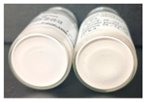		Color change
HPMCAS-LMP	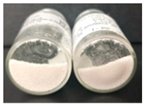		No change
HPMCAS-HMP	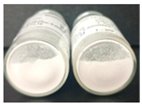		No change
HPMCAS-MMP	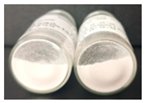		No change
HPMCP-55	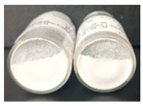		No change
Eudragit^®^ L100-55	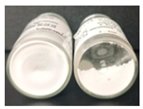		No change
Esomeprazole + HPMCAS-LMP	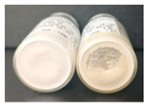		Color change
Esomeprazole + HPMCAS-HMP	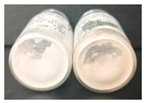		Color change
Esomeprazole + HPMCAS-MMP	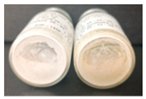		Color change
Esomeprazole + HPMCP-55	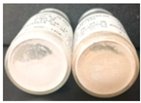		Color change
Esomeprazole + Eudragit^®^ L100-55	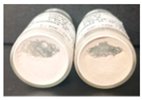		Color change

**Table 2:** Normalized spectral change descriptors and within-condition variability for the individual materials and esomeprazole–polymer mixtures after thermal stress.

System	FCI (Mean)	FCI SD	Within-Condition FCI Variability	FCI Signal/Noise Ratio	NRMSD	Relative Max Δ
Esomeprazole + HPMCP-55	0.1511	0.0235	0.0551	2.74	0.1451	0.1311
Esomeprazole + Eudragit^®^ L100-55	0.1305	0.1052	0.2226	0.59	0.1305	0.1295
Esomeprazole + HPMCAS-LMP	0.0604	0.0162	0.0487	1.24	0.0589	0.0655
Esomeprazole + HPMCAS-HMP	0.0543	0.0231	0.0430	1.26	0.0503	0.0463
Esomeprazole	0.0542	0.0130	0.0629	0.86	0.0517	0.0576
HPMCP-55	0.0482	0.0192	0.0383	1.26	0.0449	0.0462
HPMCAS-MMP	0.0464	0.0201	0.0410	1.13	0.0412	0.0484
Esomeprazole + HPMCAS-MMP	0.0463	0.0065	0.0237	1.95	0.0399	0.0312
HPMCAS-LMP	0.0405	0.0127	0.0424	0.95	0.0356	0.0352
Eudragit^®^ L100-55	0.0388	0.0109	0.0278	1.39	0.0354	0.0463
HPMCAS-HMP	0.0241	0.0078	0.0260	0.93	0.0217	0.0255

FCI, fractional change index; SD, standard deviation across the four possible reference–stress replicate pairings; NRMSD, normalized root-mean-square deviation; relative Max Δ, maximum absolute spectral difference relative to the maximum absolute reference intensity. Within-condition FCI variability was calculated as (FCI_ref_ + FCI_stress_)/2. The FCI signal-to-noise ratio was calculated as Mean FCI/Mean within-condition FCI. All metrics are descriptive because the duplicate measurements were technical replicates of the same sample preparation, not independent sample preparations.

**Table 3:** Interaction energies of the esomeprazole–polymer complexes calculated using ωB97X-D, before and after counterpoise (CP) correction for basis-set superposition error (BSSE), including zero-point energy and thermal corrections.

Complex	ΔE_int_ (kcal/mol)	ΔE_int-CP_ (kcal/mol)	E_elec+ZPE_ (kcal/mol)	E_elec+therm_ (kcal/mol)	H_elec+therm_ (kcal/mol)	G_elec+therm_ (kcal/mol)
HPMCAS-ESO	−45.33	−40.82	−38.53	−38.67	−38.89	−39.07
HPMCP-ESO	−30.87	−27.86	−24.49	−24.96	−25.04	−25.90
Eudragit^®^ L100-55-ESO	−16.32	−14.27	−11.39	−11.21	−12.80	−13.49

**Table 4:** Electronic reactivity descriptors of the polymers, esomeprazole, and esomeprazole–polymer complexes.

Molecule	HOMO	LUMO	ΔE	I	A	η	μ	χ	ω
HPMCAS-ESO	−7.946	0.342	8.288	7.946	−0.342	4.144	−3.802	3.802	0.040
HPMCP-ESO	−7.947	0.069	8.017	7.947	−0.069	4.008	−3.939	3.939	0.042
Eudragit^®^ L100-55-ESO	−8.202	−0.193	8.009	8.202	0.193	4.005	−4.197	4.197	0.048
Esomeprazole	−7.797	0.582	8.378	7.797	−0.582	4.189	−3.608	3.608	0.037
HPMCAS	−9.074	0.844	9.917	9.074	−0.844	4.959	−4.115	4.115	0.057
HPMCP	−9.052	−0.009	9.043	9.052	0.009	4.522	−4.530	4.530	0.063
Eudragit^®^ L100-55	−9.736	0.846	10.582	9.736	−0.846	5.291	−4.445	4.445	0.071

HOMO, highest occupied molecular orbital; LUMO, lowest unoccupied molecular orbital; ΔE, HOMO–LUMO energy gap; η, chemical hardness; μ, chemical potential; χ, electronegativity; ω, electrophilicity index. All energy values are expressed in eV.

## Data Availability

The raw data supporting the conclusions of this article will be made available by the authors on request.

## Supplementary Materials

The following supporting information can be downloaded at: https://www.mdpi.com/article/10.3390/polym18212655/s1, Figure S1: FTIR spectrum of HPMCAS-HMP; Figure S2: FTIR spectrum of HPMCAS-LMP; Figure S3: FTIR spectra of the esomeprazole–HPMCAS-LMP binary mixture; Figure S4: FTIR spectra of the esomeprazole–HPMCAS-HMP binary mixture.

## Abbreviations

The following abbreviations are used in this manuscript:

API	active pharmaceutical ingredient
ASD	amorphous solid dispersion
ATR	attenuated total reflectance
ATR-FTIR	attenuated total reflectance–Fourier transform infrared spectroscopy
BSSE	basis set superposition error
DFT	density functional theory
ESO	esomeprazole
FCI	fractional change index
FTIR	Fourier transform infrared spectroscopy
GERD	gastroesophageal reflux disease
HME	hot melt extrusion
HOMO	highest occupied molecular orbital
HPMCAS	hydroxypropyl methylcellulose acetate succinate
HPMCP	hydroxypropyl methylcellulose phthalate
LC–MS	liquid chromatography–mass spectrometry
LGA	Lamarckian Genetic Algorithm
LUMO	lowest unoccupied molecular orbital
MHP	maximum hardness principle
NCI	non-covalent interactions
NRMSD	normalized root-mean-square deviation
PPI	proton pump inhibitor
PVP	polyvinylpyrrolidone
RDG	reduced density gradient
Tg	glass transition temperature
