# Supplementary material for: Polymer Selection for Esomeprazole Solid Dispersions: Drug–Polymer Compatibility Assessed by Forced Degradation, ATR-FTIR, and DFT Calculations
Source: Polymers (Basel). 2026 Oct 26;18(21):2655. doi: 10.3390/polym18212655 (PMC13765801; doi:10.3390/polym18212655)
Supplement: Supplementary file 1 [file polymers-18-02655-s001.zip › polymers-4609001-supplementary.pdf]

Supplementary Materials

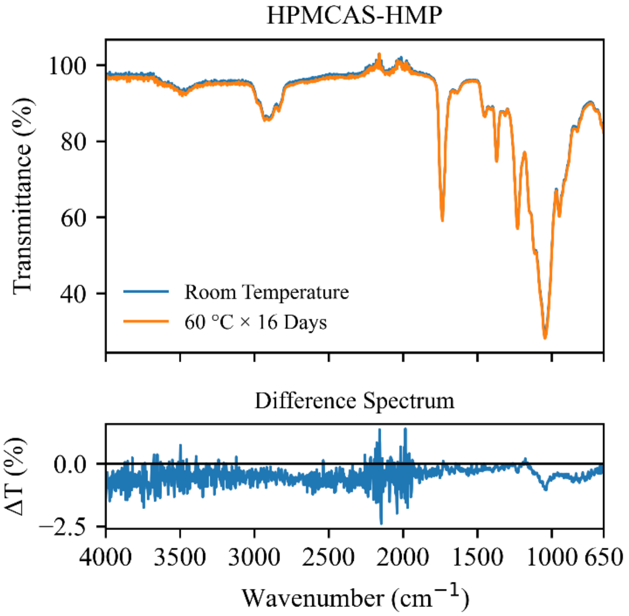

Figure S1. FTIR spectrum of HPMCAS-HMP stored at room temperature and subjected to forced degradation at 60°C for 16 days, along with its difference spectrum.

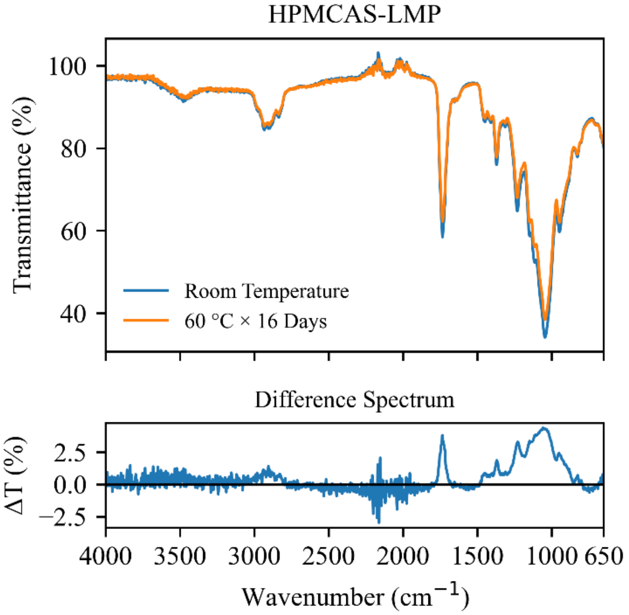

Figure S2. FTIR spectrum of HPMCAS-LMP stored at room temperature and subjected to forced degradation at 60°C for 16 days, along with its difference spectrum.

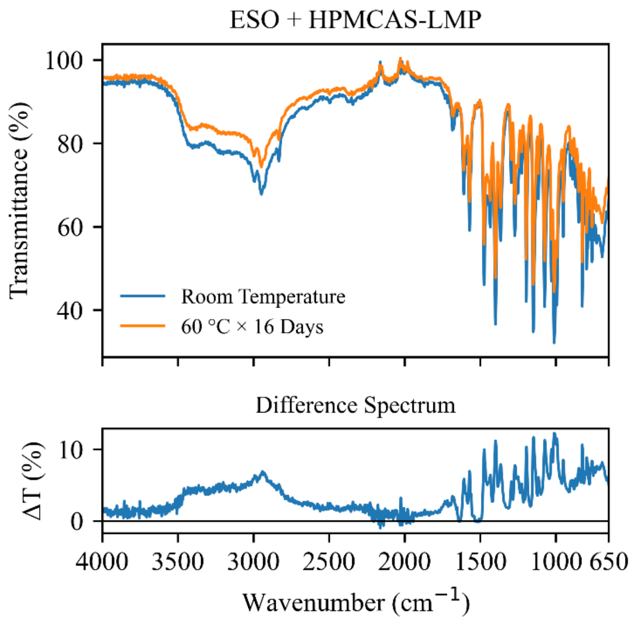

Figure S3. FTIR spectra of the esomeprazole–HPMCAS-LMP binary mixture stored at room temperature and subjected to thermal stress at 60 °C for 16 days, along with its difference spectrum.

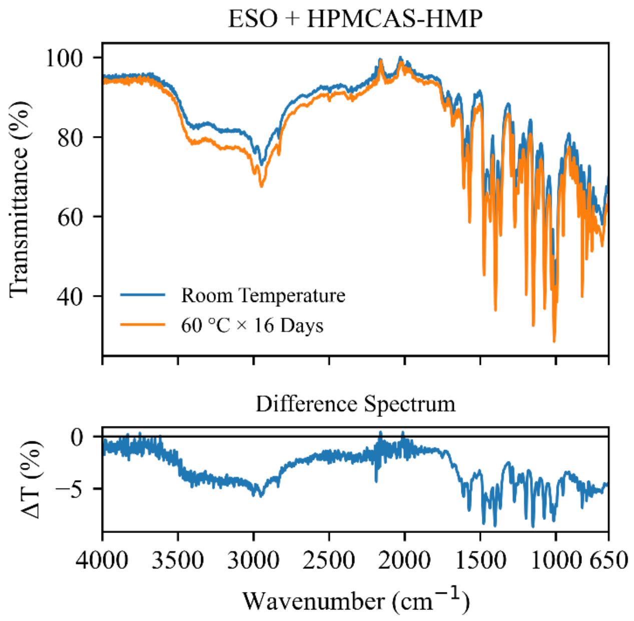

Figure S4. FTIR spectra of the esomeprazole–HPMCAS-HMP binary mixture stored at room temperature and subjected to thermal stress at 60 °C for 16 days, along with its difference spectrum.
